# The impacts of COVID-19 on routine immunization for children in Rwanda

**DOI:** 10.1186/s12879-026-12623-0

**Published:** 2026-01-24

**Authors:** Edward Mbonigaba, Fengyun Yu, Mark Donald C. Reñosa, Frederick Nchang Cho, Qiushi Chen, Wenjin Chen, Claudia M. Denkinger, Shannon A. McMahon, Simiao Chen

**Affiliations:** 1 Centre of Infectious Diseases, Division of Infectious Diseases and Tropical Medicine, Universität Heidelberg, Heidelberg, Germany; 2 College of Medicine and Health Sciences, School of Public Health, University of Rwanda, Kigali, Rwanda; 3 Interdisciplinary Centre for Scientific Computing, Universität Heidelberg, Heidelberg, Germany; 4 Heidelberg Institute of Global Health, Universität Heidelberg, Heidelberg, Germany; 5 Department of Epidemiology and Biostatistics, Research Institute for Tropical Medicine, Muntinlupa City, Philippines; 6 Cameroon Baptist Convention Health Services – HIV free/Strengthening Public Health Laboratory Systems, Kumba, Cameroon; 7 Infectious Disease Laboratory, Faculty of Health Sciences, University of Buea, Buea, Cameroon; 8 The Harold and Inge Marcus Department of Industrial and Manufacturing Engineering, The Pennsylvania State University, University Park, Harrisburg, PA United States of America; 9 School of Population Medicine and Public Health, Chinese Academy of Medical Sciences & Peking Union Medical College, Beijing, China; 10 Chinese Academy of Medical Sciences and Peking Union Medical College, Peking, China

**Keywords:** Assessment, Routine immunisation, Impact of COVID-19, Rwanda

## Abstract

**Introduction:**

The Coronavirus Disease 2019 (COVID-19) pandemic had a direct impediment to the provision of critical health services worldwide, and one of the most affected areas was routine immunisation programmes. This caused a significant drop in child immunisation rates, especially in the early stages of the pandemic. This research aimed to investigate the socio-demographic predictors of the continued persistence of routine immunisation services in Rwanda during the COVID-19 pandemic.

**Methods:**

The survey was done between January 3 and March 31, 2022 among mothers living in five districts in Rwanda. The goal of the study was to investigate issues that affect the willingness of mothers to vaccinate their children during the COVID-19 pandemic. The main outcome measure was the willingness to vaccinate children, which was divided into willing, uncertain, and unwilling. The analysis of the correlation between the maternal sociodemographic factors and the outcome variable was performed by the multinomial logistic regression model in the case when the effect of the pandemic on the attitudes to vaccination could potentially affect the outcome.

**Results:**

Two thousand four hundred and fifty-five out of the two thousand four hundred and fifty-five mothers surveyed indicated that their religion endorsed immunisation of two thousand four hundred and fifty-five mothers, 92.2% and their culture advocated immunization of two thousand four hundred and fifty-five mothers, 91.6%. The cultural and traditional support to immunization was significantly linked to the marital status, educational level, and average monthly income (*p* < 0.05). In terms of perceptions of vaccine safety, 77.3% of participants were concerned with serious adverse effects of vaccines in general, and 58.7% were in particular concerned with COVID-19 vaccinations, and only eight point 1% questioned the overall safety of COVID-19 vaccines. With the exception of age, marital status, and the number of children in the vaccination-aged group, all the other socio-demographic variables were significantly correlated with perceived risks of vaccination (*p* < 0.05).

**Conclusion:**

Our cross-sectional survey (*N* = 2,045 mothers; data collection 3 January-31 March 2022) suggests that Rwanda, like any other country in the world was affected by the pandemic. However, the shocks of the pandemic in Rwanda did not significantly affect routine immunisation because her prior investments, especially the systems and practices established during the Ebola pandemic (surveillance, infection-prevention and control [IPC], rapid risk-communication and outreach networks) could well have alleviated the harmful effect of the COVID-19 pandemic on the usual childhood immunisation as noted by World Health Organization. (2019, July 24). WHO applauds. This survey evidence indicates that pro-vaccination social norms are also strong: 92.2% of survey participants reported that their religion promotes immunisation, and 91.6% indicated that their culture promotes vaccination. These social and community support levels align with a system that emphasised the need for regular immunisation during the pandemic.

**Clinical trial:**

Not applicable.

**Supplementary Information:**

The online version contains supplementary material available at 10.1186/s12879-026-12623-0.

## Introduction

The coverage of routine immunisation before the COVID-19 pandemic (2015–2018 reported a high level of coverage of the core antigens in Rwanda. The WHO/UNICEF estimates show DTP3 coverage between 2015 and 2018 increased. However, the Coronavirus Disease 2019 (COVID-19) pandemic severely affected global health systems, overwhelming the healthcare infrastructure, diverting resources and disrupting vital health services. According to the World Health Organisation (WHO), over 90% of countries have reported having had limitations on one or more of the key health services during the early stages of the pandemic, especially the low and middle-income nations (WHO, 2021). These crises went beyond COVID-19 case control to maternal and child health care and chronic disease control and prevention care, which included vaccination programs. The most impacted services in the world included routine immunisation (RI), which is the regular, scheduled administration of vaccinations to prevent vaccine-preventable diseases (VPDs) in children and other target groups. Disruptions in RI were caused by lockdown, health facility closures, a decrease in mobility, and a redistribution of health personnel and resources to the efforts associated with responding to COVID-19. The third dose of the diphtheria-tetanus-pertussis (DTP3) vaccine coverage among children aged 1 to 18 year-olds has decreased by 81 in 2021, the largest continuous decline in childhood immunisation in 30 years (UNICEF & WHO, 2022).

Countries took some distinct measures to curb these effects such as catch-up vaccination, mobile outreach, digital health tracking, and community-based engagements to reestablish RI coverage and to rebuild societal trust in vaccination. Nonetheless, this has not led to even coverage and there has remained disparities in coverage in the sub-Saharan Africa and other low-resource environments.

In Rwanda, the resilience of the health system and previous experience in preparing against the infectious disease including Ebola virus disease (EVD) preparedness programs assisted in the reduction of the RI disruption in terms of the pandemic. The adaptive measures that were implemented to maintain the immunisation efforts through the Ministry of Health and Rwanda Biomedical Centre included integrated service delivery, enhanced surveillance systems, community health worker (CHW) participation and public health communication campaigns. Even with these measures, there were temporary falls in the coverage of vaccines, especially at the beginning of the lockdown in 2020, which is common worldwide patterns of interruption of services.

Resistance to regular immunisation amid the COVID-19 era in Rwanda was the presence of limited mobility, fear of contraction at health facilities, diversion of resources, and misinformation on vaccination. Also, misinformation that spread during the pandemic and safety fears, as well as diminished trust in health authorities, contributed to vaccine hesitancy, which was defined by WHO as the delay in level of acceptance or refusal of vaccines despite the availability of vaccination services. Research has indicated that the attitudes of parents towards the safety of vaccines, cultural and religious convictions, and socio-economic barriers contribute greatly to the issue of childhood vaccination in Rwanda.

Even though some studies have determined the overall impact of COVID-19 on health services in Rwanda, there is a paucity of evidence on how individual socio-demographic factors can determine the persistence of routine immunisation during and after the pandemic. These determinants should be understood to be used in future immunisation recovery efforts and bolster health system resilience to future outbreaks of any public health crisis.

Hence, the research question was to investigate the socio-demographic traits related to persistence of routine immunisation in mothers in Rwanda during the COVID-19 pandemic with the aim of determining variables that encourage sustained use of vaccines in the face of health system interferences.

The spread of the new Coronavirus Disease 2019 (COVID-19), caused by the virus known as Severe Acute Respiratory Syndrome Coronavirus (SARS-CoV-2), originated in Wuhan, China in December 2019 [1–3]. By 2021, the emerging variants of COVID-19 have affected around 230 million individuals globally, leading to 4.7 million deaths [4]. The number of infections surpassed 100 million people in 2020 [5, 6]. In Africa, there were 12.4 million cases and 256,000 deaths [7], with Rwanda recording 133,194 cases and 1,468 deaths [8].

One of the most common indirect impacts of the COVID-19 pandemic was the disruption of routine immunisation (RI) services [9, 10]. In Rwanda, there was a significant increase in visits to hospital by 60%, along with a corresponding decrease in visits to health centres by 15% [11, 12]. Globally, an estimated 23 million children missed routine vaccinations in 2020, marking the largest decline since 2009 and reflecting the widespread disruption of health services during COVID-19. This global setback provides context for similar declines observed in Rwanda, where routine immunization coverage temporarily dropped compared to pre-pandemic levels., 30 million missed the recommended doses of diphtheria, tetanus and pertussis (DPT3), and 27.2 million children missed the recommended doses of measles-containing-vaccine (MCV1) [10, 13–15].

In response to the pandemic, various countries adopted strategies based on their socio-demographic, cultural and political commitments [16] to maintain the continuity of routine immunisation. In March 2021, Rwanda received approximately 240,000 doses of the Oxford AstraZeneca COVID-19 vaccine, and 103,000 doses of Pfizer-BioNTech COVID-19 vaccines through the COVAX Facility platform, supported by organisations such as the Coalition for Epidemic Preparedness Innovations (CEPI), Gavi, the Vaccine Alliance, and World Health Organisation (WHO), in partnership with the United Nations Children’s Fund (UNICEF) [17–19]. The country also received a second batch of these vaccines from India in the same month of 2021 [20]. Other COVID-19 vaccines used to mitigate the pandemic were Johnson & Johnson, Moderna, Sinopharm, and Sputnik V [21, 22]. The completion of multiple doses of COVID-19 vaccines is essential for ensure a robust immune response, with vaccines like Johnson & Johnson, Oxford-AstraZeneca and Moderna requiring two doses, while for Pfizer-BioNTech may require two or three doses [18, 19, 23, 24]. Achieving a high rate of vaccination uptake and ensuring completion of the recommended doses are critical steps in mitigating the COVID-19 pandemic [25–27] and preventing other vaccine-preventable diseases. The COVID-19 pandemic brought about widespread global disruption in routine childhood immunisation especially in the initial pandemic phases [9, 10], especially with the lockdowns.There were positive and negative impacts of the rollout of the COVID-19 vaccine in Rwanda on the routine child immunization. Although it increased the country vaccination infrastructure and the population vaccination awareness, the initial interest in COVID-19 vaccination redirected the health resources and attention to the regular services. This two-prong effect demonstrates that there must be a balance between emergency responses and the sustainability of child immunization programs.

Vaccine hesitancy is influenced by various factors such as misinformation, perceived risks, and concerns about the safety of vaccines. Studies highlighted the role of anti-vaccine movements [28] and accessibility [22] issues, which impacted the continuation and completion of RI. Additionally, concerns about side effects and safety of COVID-19 vaccines further exercebated the challenge of achieving widespread vaccine coverage [23, 29, 30].

While global research has focused on vaccine acceptance, hesitance, and attitudes towards COVID-19 vaccines, there is limited literature specific to the continuation of RI in the context of the pandemic, particularly in Rwanda. Existing studies predorminantly concentrate on vaccine uptake for COVID-19 vaccines, but few explore the broader impact on routine childhood immunisation during the pandemic [31–33]. This gap is evident and leaves a critical need for more context-specific research, especially in countries like Rwanda, where socio-demographic factors such as culture, financial constraints, and vaccine misinformation may significantly influence immunisation behaviours [34, 35].

The aim of this study is to fill the gap by exploring the socio-demographic characteristics associated with the continuation of RI in Rwanda during the COVID-19 pandemic.

## Materials and methods

### Study design and participants

The research was a national cross-sectional observational research study carried out to offer evidence on the effect of the COVID-19 pandemic to routine immunization (RI) in Rwanda. The data was collected during the period between 3 rd January and 31 st March, 2022. The research design used in the study was the quantitative research design that would help to obtain data on the knowledge, attitudes, and practices of caregivers regarding child immunization during and after the COVID-19 pandemic. Mothers with the children of under five years were sampled by a structured questionnaire and sampled across the various districts of all the provinces so as to have national representativeness. The sample of the study excluded the caregivers who did not have primary roles in regard to child vaccination since it was necessary to determine the maternal views and decision-making styles having the most significant impact on RI uptake. The data analysis was conducted with the help of descriptive and inferential statistics to establish the relationships between demographic variables and immunization practices during the COVID-19 pandemic.

Rwanda is a country in the Great Lakes region of Africa, covering 26,338 square kilometres, with a population of 14.14 million people in 2021 and a growth rate of 2.38% between 2019 and 2020 [36, 37]. The median age of the Rwandan population is approximately 20 years (range: 19.5–20.4 years) [38], and the capital city, Kigali has a population of 745,261 people [37]. Although a low-income country, with over 70% of the population engaged in agriculture, Rwanda aspires to reach middle-income status by 2035 and high-income status by 2050 [37, 39].

Study participants were mothers aged ≥ 18 years who had children 12–23 months, spoke English, and provided written informed consent to participate in the study. Mothers who did not have children in the immunisation age group, male participants, and caregivers other than mothers were excluded. Data were collected from 50 villages in the selected districts.

### Sampling method and sample size calculation

A multi-stage cluster sampling method was employed. First, the five provinces were listed, and four provinces (Easter, Central, Southern, and Western) were randomly selected.

In the case of this national cross-sectional study about the effects of COVID-19 on routine immunization (RI) of children in Rwanda, the Northern Province was excluded on purpose because there were logistical and methodological factors that may influence data validity. In particular, in the study period, the Northern Province suffered significant setbacks in healthcare access due to simultaneous population health-related interventions that were not related to COVID-19, including widespread community-based health initiatives on malaria and maternal-child health. This province would have confounded others since changes in immunization cover in this region may be due to these common interventions and not necessarily due to the actual influence of the COVID-19 pandemic. Thus, to achieve methodological rigor and less bias in estimating the specific impact of the pandemic on RI, the study concentrated on the four remaining provinces with stable and similar systems of routine immunization services and data collections during the study period. This method is in line with the general epidemiological procedures, where areas of distinct confounding effects are omitted in a bid to maintain internal validity at the expense of a representative measure of national trends (Kadam & Bhalerao, 2010).

**Fig. 1:**
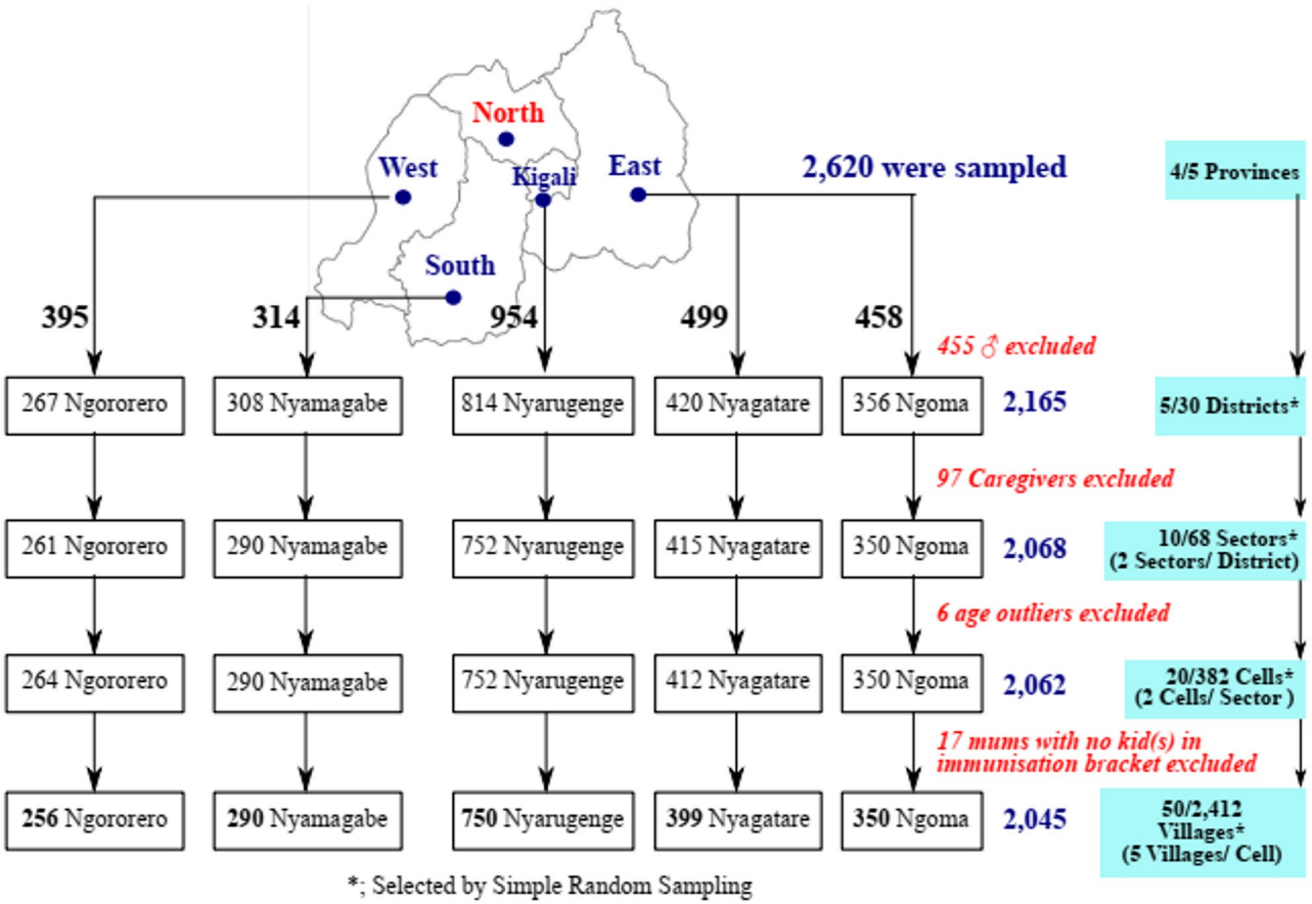
Schematic illustration of the study design, settings, and eligibility of respondents

One distict was randomly selected from each province, and within each district, two sectors were selected. For each sector, two “Akagalis” (villages) were chosen. A total of 50 villages in five health districts of Rwanda were chosen for data collection (Fig. 1).

In each selected village, mothers were consecutively and conveniently recruited intil the desired sample size was achieved. In the absence of similar studies in Rwanda, a minimum sample size of 154 mothers per Cluster/District, based on the WHO immunisation coverage cluster survey [40], was calculated with the CDC Epi Info version 7.2.5.0 (Centre for Disease Control, Georgia, USA) StatCalc with the following characteristics: an estimated District population size of 362,806 in 2022 [37], an estimated proportion of mothers who continued routine immunisation after the emergence of the COVID-19 pandemic of 50.0%, a design effect of 2.0, an accepted error margin of 5%, and five Districts. Considering possible non-response and non-responding respondents, the sample size was adjusted by 10% (16 respondents) to 170.

### Definition of concepts and study variables

#### Independent and demographic variables

Socio-demographic variables included age, religion, marital and educational status, number of children in the immunisation bracket, occupation, and average monthly income. Other independent variables were religion and culture (Q23–24; S1 Appendix), and lack of funds (Q25; S1 Appendix).

#### Outcome variables

The primary outcome was the effect of the COVID-19 pandemic on the willingness to get children vaccinated. Other outcome variables included the effect of religion, culture, economic status and perception of risk on the children’s routine vaccination and the degree of risk perception, misinformation and trust. The questions and coding schemes of the above outcome variables are outlined in Table 1.

**Table 1:** Question for outcome variables

Index	Category	Question	Response(s)	Coding
XX	Primary outcome	Has the COVID-19 pandemic affected your routine immunisation of your children?	- Yes; - No	- Yes = 0; - No = 1
Q23	Religion	Does your religion support the Immunisation of children?	- Yes; - No	- Yes = 0; - No = 1
Q24	Culture	Does your culture support the Immunisation of children?	- Yes; - No	- Yes = 0; - No = 1
Q25	Economic status	Does lack of funds ever prevent you from Immunisation of your child?	- Yes; - No; - I don’t know	- Yes = 1; - No = 0; - I don’t know1
Q26	Perception of risk	Does the message you hear or receive from anti-vaccine groups or propaganda affect your confidence in the vaccination of your children?	- Yes; - No	- Yes = 0; - No = 1
Q27	Trust/Confidence	Do you trust the information you receive about the vaccination of your children?	- Yes; - No	- Yes = 0; - No = 1
Q38	Perception of risk	I am concerned about the serious adverse effects of vaccines.	- Strongly Disagree, - Disagree, - Undecided/Neutral, - Agree, - Strongly Agree	- **Disagree**; Strongly Disagree, Disagree, Undecided/Neutral, - **Agree**; Agree, Strongly Agree
Q50	Perception of risk	I am concerned about the serious adverse effects of COVID-19 vaccines	- Strongly Disagree, - Disagree, - Undecided/Neutral, - Agree, - Strongly Agree	- **Disagree**; Strongly Disagree, Disagree, Undecided/Neutral, - **Agree**; Agree, Strongly Agree
Q53	Perception of risk	I am concerned that COVID-19 vaccines might not be safe.	- Strongly Disagree, - Disagree, - Undecided/Neutral, - Agree, - Strongly Agree	- **Disagree**; Strongly Disagree, Disagree, Undecided/Neutral, - **Agree**; Agree, Strongly Agree
Q56	Misinformation	Received negative information about COVID-19 vaccines	- Yes; - No	- Yes = 0; - No = 1
Q57	Trust/Confidence	The trust in negative information on the COVID-19 vaccine	- Yes; - No	- Yes = 0; - No = 1

VA; Vaccine Acceptance, VH; Vaccine Hesitancy

The potential responses to each of these questions were sorted on a Likert scale [41] and included Strongly disagree, Disagree, Undecided, Agree, and Strongly agree. These potential responses were then scored as one, two, three, four, and five, respectively. Modified Bloom’s cut-off points were used to rate the perception of risk of vaccination as very poor (< 20%), poor (≥ 20 but < 40%), moderate (≥ 40 but < 60%), good (≥ 60 but < 80%), or very good (≥ 80%) and later as poor (< 60%) or good (≥ 60%) [42, 43].

### Sampling method

We implored a multistage Cluster (province) sampling method where a list of all Cells/“Akagalis” and villages therein was drawn. A total of 50 villages were selected, including at least two villages from each Cell/“Akagali”. The sampling procedure for the required number of mothers was done in three stages.

Firstly, the five provinces were listed and then four; the Western, Southern, Centre/Kigali, and Eastern provinces were selected by using random proportions of the RAN function in Microsoft Office Excel, 2016 (Microsoft Corporation Inc. USA), wherein the least proportion attributed to the Northern province was not selected. Secondly, the number of districts of the selected provinces was listed, and one of each was selected using Simple Random Sampling (SRS). Within each district, two sectors were selected and within each sector, two Akagalis were selected. At least two villages were then selected from each Akagali by SRS (Fig. 1). Thirdly, within each selected village, mothers were sampled conveniently and consecutively until the desired sample size was attained or surpassed. The convenience and consecutive sampling techniques were applied wherein participants were approached and informed of the study objectives at their workplaces and doorsteps. Data was then collected through personal interviews.

### Data collection

Data was collected using well-structured questionnaires in face-to-face interviews, which to 20–30 min to complete. The questionnaire, prepared in English and translated into Kinyarwanda language to collect information on respondents’ identification, demographic characteristics (age, religion, marital status, occupation, average monthly income), information about child(ren) immunisation status, RI, the perception of risks and others. The validity of the questionnaire was confirmed by pre-testing in 10 participants who were excluded from the analysis. Based on the pre-test study, the format and wording of some questions were refined. The data obtained from the 10 participants was used to assess internal consistency reliability using Cronbach’s alpha (α) [44–46]. The results showed adequate internal consistency reliability (with Cronbach’s α = 0.72) [45, 46] for the eight sections with 62 questions.

### Justification for pre-testing 10 participants in our study

The pilot test, which was used in this research work, was deemed sufficient and appropriate due to a number of reasons.Methodologically for example, Hertzog (2008) notes that 10–15 respondents will be enough to find out the significant issues in questionnaire design, flow, comprehension, and administration processes. Second, 10 participants is the minimum value of 10–30 range of pilot studies generally recommended in health and epidemiology studies, especially when the target population is relatively homogeneous as was the case in the present study of routine childhood immunisation services in Rwanda. The decision to raise the pilot percentage to 10 participants, consequently, is a methodologically conservative choice, which will increase the chance of finding ambiguities or logistical flaws without wasting resources. Lastly, the pilot data were not included in the actual analysis, which meant that the internal validity of the final study was not jeopardised. Therefore, the sample size of 10 individuals instead of 9% is appropriate according to the best practices in pilot testing. At the end of each day or after every two days of field data collection, the data quality control officers checked the entry of all data into the Microsoft Office Excel sheet to ensure that the right data was being collected. Data was reviewed and sorted multiple times to removed duplicated data before exported to a Microsoft Office Excel sheet. At the end of the data collection exercise, the field supervisor checked all the data from the various districts to ensure that the data collected was in order.

### Variables and measurements

#### Covariates and outcomes

Age groups, religion, marital status, educational status, number of child(ren) in the immunisation bracket, occupation and average monthly income group were summarised as counts and percentages. Multivariate/multinomial logistic regression was also used to determine associations between the outcome variables (effect of COVID-19 on routine immunisation of children/dependent variables with demographic characteristics.

Multicollinearity was tested for, and the following models were used:

Culture supports the immunisation of children = β_0_ + β_1_Age Group + β_2_Religion + β_3_Marrital Status + β_4_Education + β_5_Number of children in the immunisation bracket + β_6_Occupation + β_7_Monthly Income + ε,

Religion supports the immunisation of children = β_0_ + β_1_Age Group + β_2_Religion + β_3_Marrital Status + β_4_Education + β_5_Number of children in the immunisation bracket + β_6_Monthly Income + ε.

Good perception of vaccine risks = β_0_ + β_1_Age Group + β_2_Religion + β_3_Marrital Status + β_4_Education + β_5_Number of children in the immunisation bracket + β_6_Occupation + β_7_Monthly Income + ε, and.

Anti-vaccine misinformation/Trust of misinformation = β_0_ + β_1_Age Group + β_2_Religion + β_3_Marrital Status + β_4_Education + β_5_Number of children in the immunisation bracket + β_6_Occupation + β_7_Monthly Income + ε. and.

Effects of COVID-19 on RI = β0 + β1Culture + β2Lack of funds + β3Vaccine misinformation + β4Trust of vaccine misinformation + β5Concerned about AEs of vaccines + β6Concerned about AEs of COVID-19 vaccines + β7Concerned that COVID-19 vaccines might not be safe + ε.

Where β_0_ is a constant, β_1_, β_2_, β_3_, β_4_, β_5_, β_6_, and β_7_ are coefficients and ε is the regression error. For multicollinearity, a variance inflation factor between 1 and 5 indicated a moderate correlation between a given predictor variable and other predictor variables in the model [47]. The significance level was set at 0.05. Data were analysed using CDC Epi Info version 7.2.5.0 (Centre for Disease Control, Georgia, USA).

### Ethical consideration

This study was conducted in strict accordance with the Declaration of Helsinki [48] and approved by the Institutional Review Board (IRB) of the University of Rwanda, College of Medicine and Health Science (No. 402/CMHS IRB/2020) and the Ethics Committee of Universität Heidelberg, Germany (S-829/2021). All study participants signed the informed consent before being interviewed. All participants were informed and assured that the data collected would be used only for research purposes and that their responses would not be available to the public.

### Anonymity and confidentiality were guaranteed

The anonymity and confidentiality of the participants were observed during the study. No personal data was taken, and all answers were coded in a way that did not allow tracking of personal participants. Information was kept in a safe place and could only be accessed by the research team. The results have been given in an aggregated form in order to safeguard the identity of all the respondents.

## Results/ statistical analysis

### Characteristics of study population

Socio-demographic characteristics are summarised in Table 2. A total of 2,620 respondents were surveyed, from which 2,045 (78.2%) mothers were included in these analyses as follows: 350 (17.1%) from Ngoma, 256 (12.5%) from Ngororero, 399 (19.5%) from Nyagatare, 290 (14.2%) from Nyamagabe, and 750 (36.7%) from Nyarugenge.

Amongst these participants, [1,072 (52.4%, 95%C.I; 50.3–54.6)] were 30 years or less [mean age of 30.6 years (SD 6.3, range 18–56)], about two-fifths [809 (39.6%, 95%C.I; 37.5–41.7)] had attained a secondary level of education, about three-quarters [1,538 (75.2%, 95%C.I; 73.3–77.0)] earned less than one United States Dollar (USD) per month, and about three-quarters [1,586 (77.6%, 95%C.I; 75.7–79.3)] were married (Table 2).

Bivariate analysis revealed that, except for the number of child(ren) in the immunisation bracket, all the other characteristics of the study participants were associated with the Districts (Table 2).

### Sources of child vaccination information

A large majority of the mothers (94.1%, 95%C.I; 93.0–95.1) had vaccine information from healthcare workers (Medical Doctors and Nurses), about a quarter (28.8%, 95%C.I; 26.9–30.8) from the Mass media (Radio and Television), and a very minute (7.1%, 95%C.I; 6.1–8.3) proportion from relationships (friends, co-workers, and neighbours) (Supplementary Table 1).

### Reasons for vaccination/immunisation discontinuation

A total of 1,781 participants (96.6%, 95% C.I; 95.6–97.3) reported that they had no problem with taking their children for vaccination. Among the participants who did not take their children for vaccinations, only 122 (5.5%; 95% C.I; 4.6–6.6) cited inaccessibility to healthcare facilities and 44 (2%, 95% C.I; 1.5–2.7) indicated that they do not believe that vaccines are good for their children as reasons.

### The multicollinearity results

*Prior to multivariate analysis*,* the multicollinearity of the independent variables that were included in the regression models was measured. All covariates connected to socio-demographic features*,* the accessibility of health services*,* and the COVID-19-related disruptions were tested on variance inflation factors (VIFs) and tolerance values. As a result*,* the entire range of covariates was included in the resulting multivariate regression models*,* and the estimation of the relationships between COVID-19-related variables and the outcomes of routine immunisation was deemed to be consistent and accurate*.

**Table 2:** Socio-demographic characteristics of study participants (*n* = 2,045)

Variable	Subclass	Total (%)	Percent (95% C.I)	District					χ^2^ (F)	*p*-value
				Ngoma	Ngororero	Nyagatare	Nyamagabe	Nyarugenge		
Age (in years)	≤ 30	1,072 (52.4)	52.4 (50.3–54.6)	186	116	253	151	383	25.835	**1.1 × 10**^**− 4**^
	31–40	874 (42.7)	42.7 (40.6–44.9)	139	119	149	131	336		
	**≥** 41	99 (4.8)	4.8 (4.0–5.9)	45	21	15	8	30		
	Mean age ($$\bar{\mathrm{x}}$$ ± SD)	30.6 ± 6.3		30.3 ± 6.6	31.7 ± 6.5	29.4 ± 6.2	30.6 ± 5.8	30.9 ± 6.1	6.666	**< 0.001**
Religion	Catholic	85 (4.2)	4.2 (3.4–5.1)	6	1	8	22	48	30.029	**< 0.001**
	Protestant	1,960 (95.8)	95.8 (94.9–96.6)	344	255	391	268	702		
Marital status	Divorced	59 (2.9)	2.9 (2.2–3.7)	5	1	11	10	32	31.296	**1.8 × 10** ^**− 3**^
	Married	1,586 (77.6)	77.6 (75.7–79.3)	269	199	315	213	590		
	**S**ingle	373 (18.2)	18.2 (16.6–20.0)	71	52	71	57	122		
	Widowed	27 (1.3)	1.3 (0.9–1.9)	5	4	2	10	6		
Education	NFE	190 (9.3)	9.3 (8.1–10.6)	48	16	45	63	18	522.405	**< 0.001**
	Primary	963 (47.1)	47.1 (44.1–49.3)	251	192	218	106	196		
	Secondary	809 (39.6)	39.6 (37.5–41.7)	48	45	130	112	474		
	Tertiary	83 (4.1)	4.1 (3.3–5.0)	3	3	6	9	62		
# of child(ren)	1	1,969 (96.3)	96.3 (95.4–97.0)	335	246	380	286	722	5.989	2 × 10^− 1^
	2–4	76 (3.7)	3.7 (2.9–4.6)	15	10	19	4	28		
	Mean child(ren) ($$\bar{\mathrm{x}}$$ ± SD)	1.04 ± 0.2		1.05 ± 0.3	1.06 ± 0.3	1.05 ± 0.2	1.03 ± 0.3	1.04 ± 0.2	0.772	5.4 × 10^− 1^
Occupation	Artisan	1,143 (55.9)	55.9 (53.7–58.0)	332	212	258	265	76	1,133.841	**< 0.001**
	Casual labour	192 (9.4)	9.4 (8.2–10.7)	4	11	13	22	142		
	Civil Servant	110 (5.4)	5.4 (4.5–6.4)	2	14	30	0	64		
	Unemployed	600 (29.3)	29.3 (27.4–31.4)	12	19	98	3	468		
AMI (in $)	< 100	1,538 (75.2)	75.2 (73.3–77.0)	240	255	356	273	414	396.124	**< 0.001**
	101–200	193 (9.4)	9.4 (8.3–10.8)	43	1	36	17	96		
	201–300	167 (8.2)	8.2 (7.1–9.4)	37	0	6	0	127		
	> 400	147 (7.2)	7.2 (6.2–8.4)	30	0	1	0	116		
Total (%)		2,045	100.0	350 (17.1)	256 (12.5)	399 (19.5)	290 (14.2)	750 (36.7)		

1$ = 1.161 RWF = 0.912 € [49], #; number, %; proportion of respondents, $$\bar{\mathrm{x}}$$; Mean, 95% C.I; 95% Confidence interval, AMI; Average Monthly Income, Boldface numbers indicate significant *p* values, F; F-Statistic (Fisher test), NFE; No Formal Education, SD; Standard Deviation

On the anticipated most important reasons for the non-completion of vaccination doses, 99 (4.8%, 95% C.I; 4.0–5.8) of the mothers asserted that the child was healthy, 99 (4.8%, 95% C.I; 4.0–5.8) were sceptical by indicating that they were not sure the vaccines were good for their children, while four (0.2%, 95% C.I; 0.08–0.5) stated that neighbours children suffered complications from vaccination shots (Supplementary Table 2).

In a separate question, the mothers were asked; ‘Does lack of funds ever prevented you from immunisation of your child’, and only 198 (9.7%, 95% C.I; 8.5–11.0) of them said ‘yes’. A hundred and forty-eight (74.7%) of the 198 were artisan workers, and 185 (93.4%) earned less than $100 in a month. The lack of funds or transport to the health facility was significantly associated with the occupation and average monthly income (Supplementary Table 3).

### Religious and traditional tendencies towards the immunisation of children

Of the 2,045 mothers, 1,886 (92.2%, 95% C.I; 91.0–93.3) and 1,873 (91.6%, 95% C.I; 90.3–92.7) admitted respectively that their religion and their culture supported the immunisation of children.

Logistic regression analysis revealed that marital status, educational status, and monthly income were significantly associated with, ‘Does religion’ and ‘Does culture’ support the immunisation of children? From the regression analysis, the odds for religion vs. culture to support the immunisation of children was higher amongst divorcees (*p* = 4.5 × 10^− 1^, aOR; 1.2, 95%C.I; 0.7–2.3) vs. (*p* = 7.3 × 10^− 1^, aOR; 1.1, 95%C.I; 0.6–1.9) when compared with married women, singles, and widows (Table 3).

**Table 3:** Multivariable logistic regression of demographic predictors of religious and traditional tendencies towards routine immunisation (RI)

Characteristic	Religion (*n* = 1,886)				Culture (*n* = 1,873)			
	*n* (%)	*p*-value	OR (95% C.I)	aOR (95% C.I)	*n* (%)	*p*-value	OR (95% C.I)	aOR (95% C.I)
Age (in years)								
31–40/ ≤ 30	814/ 981	**4.0 × 10** ^**− 4**^	0.7 (0.5–0.9)	0.7 (0.6–0.8)	802/ 979	6.7 × 10^− 1^	0.9 (0.6–1.3)	0.9 (0.8–1.2)
≥ 41/ ≤ 30	91 (4.8)	9.8 × 10^− 1^	0.9 (0.4–1.9)	†1.0 (0.6–1.5)	92 (4.9)	7.9 × 10^− 1^	0.8 (0.3–1.9)	0.9 (0.6–1.5)
Religion								
Protestant/Catholic	1,811/ 75	1.1 × 10^− 1^	0.6 (0.3–1.3)	0.7 (0.5–1.1)	1,801/ 72	**1.0 × 10** ^**− 2**^	0.3 (0.3–1.0)	0.6 (0.4–0.9)
Marital status								
Single/Married	358/ 1,445	**5.0 × 10** ^**− 4**^	0.4 (0.2–0.6)	0.4 (0.3–0.5)	356/ 1,435	**5.1 × 10** ^**− 3**^	0.5 (0.3–0.8)	0.5 (0.4–0.7)
Divorced/Married	56 (2.9)	4.9 × 10^− 1^	†1.1 (0.3–3.7)	†1.2 (0.7–2.3)	55 (2.9)	7.3 × 10^− 1^	†1.1 (0.4–3.5)	†1.1 (0.6–1.9)
Widowed/Married	27 (1.4)	9.8 × 10^− 1^	0.03 (0.01–0.05)	0.03 (0.01–0.04)	27 (1.4)	9.6 × 10^− 1^	0.02 (0.01–0.04)	0.02 (0.01–0.05)
Education								
Secondary/Tertiary	732/ 79	**3.0 × 10** ^**− 3**^	0.4 (0.1–1.6)	0.4 (0.2–0.7)	725/ 77	**8.3 × 10** ^**− 4**^	0.2 (0.1–0.6)	0.1 (0.05–0.2)
Primary/Tertiary	899 (47.7)	**3.0 × 10** ^**− 4**^	0.3 (0.1–1.0)	0.3 (0.1–0.5)	895 (47.8)	**1.0 × 10** ^**− 4**^	0.1 (0.02–0.3)	0.04 (0.02–0.08)
NFE/Tertiary	176 (9.3)	**1.5 × 10** ^**− 3**^	0.3 (0.1–1.2)	0.3 (0.1–0.6)	176 (9.4)	**2.0 × 10** ^**− 4**^	0.1 (0.02–0.3)	0.03 (0.02–0.07)
# of children [2–4/ 1]	74/ 1,812	**5.8 × 10** ^**− 2**^	0.4 (0.1–1.6)	0.5 (0.2–1.0)	75/ 1,798	1.8 × 10^− 1^	0.2 (0.03–1.5)	0.3 (0.1–0.8)
Occupation								
Artisan/Civil Servant	1,021/ 110	/	/	/	1,011/ 109	**3.0 × 10** ^**− 4**^	†7.0 (0.9–53.7)	†14.1 (3.5–59.6)
Casual labour/Civil Servant	184 (9.8)	/	/	/	181 (9.7)	**2.9 × 10** ^**− 3**^	†6.2 (0.7–51.7)	†9.9 (2.3–42.8)
Unemployed/Civil Servant	571 (30.3)	/	/	/	572 (30.5)	4.5 × 10^− 1^	†1.8 (0.2–14.2)	†1.7 (0.4–7.4)
AMI (in $)								
101–200/ < 100	185/ 1,389	**2.7 × 10** ^**− 3**^	0.3 (0.1–0.7)	0.3 (0.2–0.5)	182/ 1,377	**2.1 × 10** ^**− 3**^	0.3 (0.1–0.6)	0.2 (0.1–0.3)
201–300/ < 100	167 (8.8)	9.2 × 10^− 1^	0.02 (0.01–0.06)	0.02 (0.01–0.05)	167 (8.9)	9.0 × 10^− 1^	0.03 (0.02–0.06)	0.03 (0.02–0.05)
> 400/ < 100	145 (7.7)	**1.0 × 10** ^**− 3**^	0.1 (0.07–0.3)	0.07 (0.03–0.2)	147 (7.8)	9.0 × 10^− 1^	0.04 (0.01–0.07)	0.04 (0.01–0.06)
CONSTANT		2.1 × 10^− 1^				3.6 × 10^− 1^		

Legend. #; number, %; proportion of respondents, †; Most likelihood category, 95% C.I; 95% Confidence interval, AMI; Average Monthly Income, aOR; Adjusted odds ratio, Boldface numbers indicate significant *p*-values, *n*; frequency/count, NFE; No Formal Education, OR; Odds ratio, Reference Category; Yes

### Mothers’ perception of risks

**Fig. 2:**
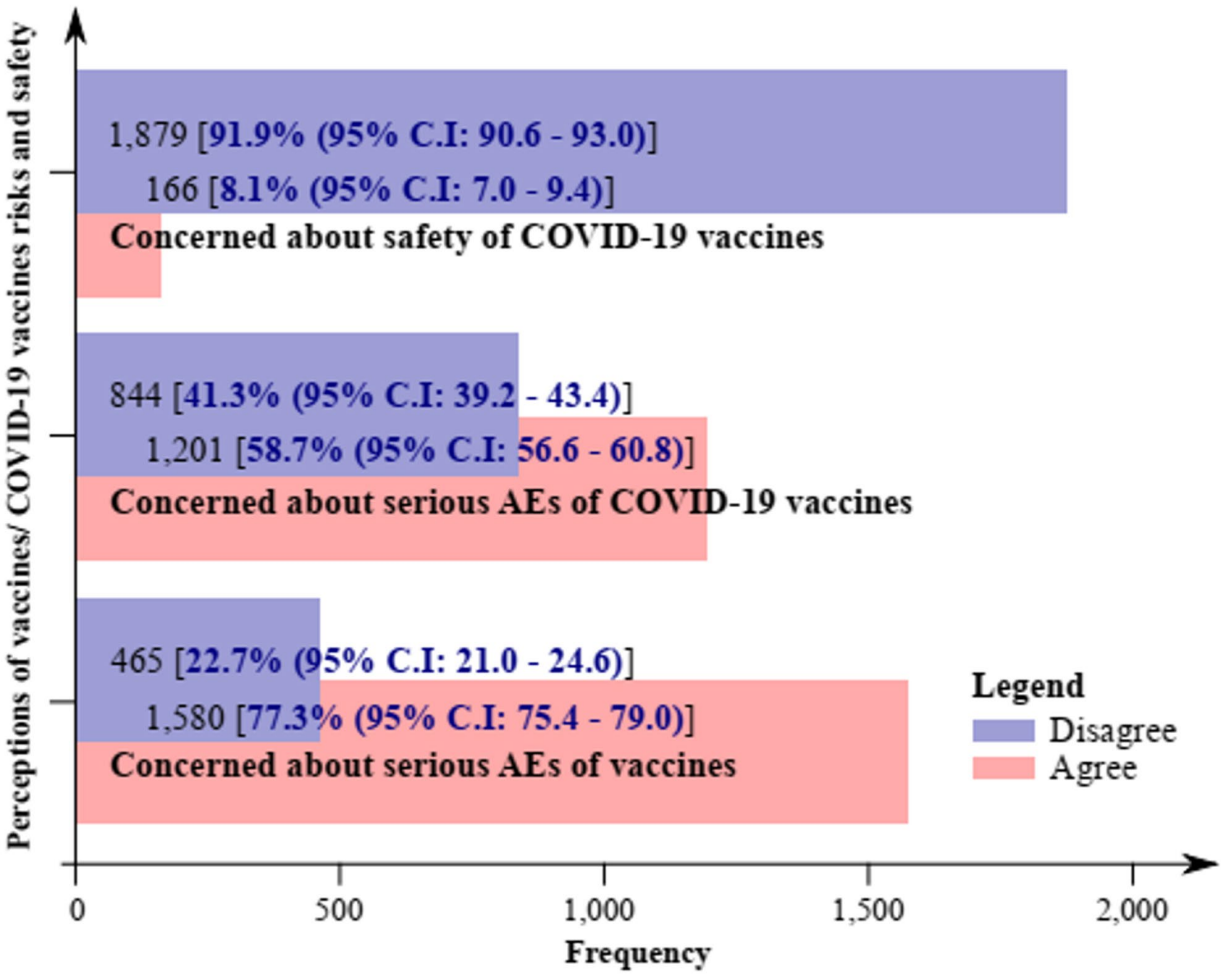
Perceptions of vaccines/COVID-19 vaccines risks and safety

Of the 2,045 mothers in the study, 1,580 (77.3%, 95% C.I; 75.3–79.0), 1,201 (58.7%, 95% C.I; 56.6–60.8), and 166 (8.1%, 95% C.I; 7.0–9.4) of the mothers agreed to the fact that; there were serious adverse effects (AEs) of vaccines, serious AEs of COVID-19 vaccines, and that the COVID-19 vaccines may not be safe respectively. Less than one-quarter [465 (22.7%, 95% C.I: 21.0–24.6)] of the respondents disagreed on the existence of serious AEs of vaccines, while 844 (41.3%, 95% C.I; 39.2–43.4) of them disagreed the existence of serious AEs of COVID-19 vaccines as well as a large majority [1,879 (91.9%, 95% C.I: 90.6–93.0)] disagreed on the fact that COVID-19 vaccines may have safety concerns (Fig. 2).

The perception of vaccine risks and safety by respondents was obtained from the summing up together of; ‘I am concerned about the serious AEs of vaccines’, ‘‘I am concerned about the serious AEs of COVID-19 vaccines’, and ‘I am concerned about the safety of COVID-19 vaccines’. Thus, for the perception of vaccine risks, 1,276 (62.4%, 95% C.I; 60.3–64.5) had a good perception; mean vaccine perception score of 61.2 (SD 14.1, range 20.0–100.0).

Bivariate and multivariate logistic regression analyses revealed that, except age, and marital status, all the socio-demographic characteristics were significantly associated with the perceived risks of vaccination. From the multivariate regression analysis, the odds for having a good risk perception of vaccines were higher amongst mothers of ≥ 41 years (*p* = 2.1 × 10^− 1^, OR; 1.2, 95%C.I; 0.8–1.7), protestants (*p* = 1.7 × 10^− 2^, aOR; 2.5, 95%C.I; 1.6–3.9), divorcees (*p* = 9.3 × 10^− 1^, aOR; 1.1, 95%C.I; 0.7–1.5), primary school leavers (*p* = 6.3 × 10^− 3^, aOR; 2.1, 95%C.I; 1.2–3.6) as well as those with NFE (*p* = 1.0 × 10^− 4^, aOR; 5.1, 95%C.I; 2.8–8.9), when compared with their various social groups =; mothers between 31 and 40 years old, Catholic Christians, married mothers and secondary vs. tertiary school leavers (Table 4).

**Table 4:** Perception of vaccine risks

S/*N*	Characteristic	Good perception (*n* = 1,276)				
		*n* (%)	χ^2^ (*p*-value)	*p*-value	OR (95% C.I)	aOR (95% C.I)
1.	**Age (in years)**					
	31–40/ ≤ 30	548/ 678	6.331 (**4.2 × 10**^**− 2**^)	6.7 × 10^− 1^	1.0 (0.8–1.2)	1.0 (0.8–1.2)
	≥ 41/ ≤ 30	50 (3.9)		2.1 × 10^− 1^	†1.3 (0.8–2.2)	†1.2 (0.8–1.7)
2.	**Religion**					
	Protestant/Catholic	1,202/ 74	21.907 (**2.9 × 10**^**− 6**^)	**1.7 × 10** ^**− 2**^	†2.4 (1.2–4.8)	†2.5 (1.6–3.9)
3.	**Marital status**					
	Single/Married	222/ 996	6.437 (9.2 × 10^− 2^)	6.2 × 10^− 1^	1.0 (0.7–1.3)	1.0 (0.8–1.1)
	Divorced/Married	44 (3.4)		9.3 × 10^− 1^	0.6 (0.3–1.3)	†1.1 (0.7–1.5)
	Widowed/Married	14 (1.1)		**2.9 × 10** ^**− 3**^	0.7 (0.3–1.6)	0.6 (0.3–0.9)
4.	**Education**					
	Secondary/Tertiary	644/ 71	231.220 (**< 0.001**)	6.9 × 10^− 1^	†1.3 (0.6–3.0)	0.9 (0.5–1.5)
	Primary/Tertiary	494 (38.7)		**6.3 × 10** ^**− 3**^	†3.1 (1.3–6.9)	†2.1 (1.2–3.6)
	NFE/Tertiary	67 (5.3)		**1.0 × 10** ^**− 4**^	†5.7 (2.3–13.7)	†5.1 (2.8–8.9)
5.	**# of children [2–4/ 1]**	50/ 1,226	0.252 (6.2 × 10^− 1^)	4.2 × 10^− 1^	0.9 (0.5–1.7)	0.8 (0.5–1.3)
6.	**Occupation**					
	Artisan/Civil Servant	477/ 79	486.781 (**< 0.001**)	**1.0 × 10** ^**− 4**^	0.8 (0.5–1.6)	0.5 (0.3–0.7)
	Casual labour/Civil Servant	172 (13.5)		**< 0.001**	0.2 (0.1–0.5)	0.2 (0.1–0.3)
	Unemployed/Civil Servant	548 (42.9)		**< 0.001**	0.06 (0.03–0.1)	0.03 (0.02–0.04)
7.	**AMI (in $)**					
	101–200/ < 100	138/ 878	81.611 (**< 0.001**)	**2.5 × 10** ^**− 2**^	0.6 (0.4–0.9)	0.4 (0.3–0.5)
	201–300/ < 100	135 (10.6)		**< 0.001**	0.3 (0.2–0.5)	0.1 (0.08–0.2)
	> 400/ < 100	125 (9.8)		**< 0.001**	0.3 (0.2–0.5)	0.05 (0.03–0.09)
	CONSTANT			**6.5 × 10** ^**− 2**^		

Legend. #; number, %; proportion of respondents, †; Most likely category, 95% C.I; 95% Confidence interval, AMI; Average Monthly Income, aOR; Adjusted odds ratio, Boldface numbers indicate significant *p*-values, *n*; frequency/count, NFE; No Formal Education, OR; Odds ratio, Reference Category; Good perception, **χ**^**2**^; Pearson Chi-Square

### Vaccine misinformation

Generally, 379 (18.8%, 95% C.I; 17.2–20.6) of the mothers respectively admitted that messages from anti-vaccine groups affect their confidence in vaccinating their children.

Logistic regression analysis revealed that age, marital status, and monthly income of the mothers were significantly associated with, misinformation concerning vaccines. Age, marital status, and monthly income were also found to be significantly associated with trust in misinformation of vaccines. =.

Multinomial regression analysis showed that the information and misinformation about vaccines were among different social, cultural and religious responders. Some categories showed statistically higher odds ratios while others showed no significant association, as described in Table 5).

**Table 5:** Multivariable logistic regression of demographic predictors of anti-vaccine misinformation vs. trust of misinformation

S/*N*	Characteristic	Anti-vaccine misinformation (*n* = 379)				Trust of misinformation (*n* = 1,968)			
		*n* (%)	*p*-value	OR (95% C.I)	aOR (95% C.I)	*n* (%)	*p*-value	OR (95% C.I)	aOR (95% C.I)
1.	**Age (in years)**								
	31–40/ ≤ 30	173/ 181	**2.6 × 10** ^**− 3**^	0.8 (0.6–1.0)	0.8 (0.7–0.9)	845/ 1,027	**3.7 × 10** ^**− 3**^	0.7 (0.4–1.1)	0.6 (0.5–0.8)
	≥ 41/ ≤ 30	25 (6.6)	**1.0 × 10** ^**− 5**^	0.5 (0.3–0.9)	0.4 (0.3–0.5)	96 (4.9)	1.6 × 10^− 1^	0.5 (0.2–1.9)	0.5 (0.3–1.2)
2.	**Religion**								
	Protestant/Catholic	353/ 26	**1.0 × 10** ^**− 5**^	†1.9 (1.2–3.1)	†1.7 (1.4–2.3)	1,885/ 83	7.1 × 10^− 1^	†1.1 (0.3–5.0)	†1.2 (0.5–2.5)
3.	**Marital status**								
	Single/Married	63/ 291	2.7 × 10^− 1^	0.8 (0.6–1.2)	0.9 (0.7–1.1)	361/ 1,522	**2.6 × 10** ^**− 2**^	0.6 (0.3–1.2)	0.6 (0.4–0.9)
	Divorced/Married	18 (4.7)	**8.6 × 10** ^**− 3**^	0.5 (0.3–1.0)	0.6 (0.5–0.9)	59 (3.0)	9.5 × 10^− 1^	0.03 (0.01–0.05)	0.03 (0.02–0.05)
	Widowed/Married	7 (1.8)	1.1 × 10^− 1^	0.6 (0.2–1.5)	0.6 (0.4–1.1)	26 (1.3)	1.8 × 10^− 1^	0.6 (0.07–4.7)	0.3 (0.09–1.6)
4.	**Education**								
	Secondary/ Tertiary	158/ 17	2.6 × 10^− 1^	†1.3 (0.6–2.6)	†1.2 (0.8–1.8)	785/ 82	3.2 × 10^− 1^	†1.2 (0.1–12.8)	0.5 (0.1–2.0)
	Primary/ Tertiary	173 (45.6)	8.2 × 10^− 1^	†1.5 (0.7–3.1)	†1.0 (0.7–1.8)	923 (46.9)	4.2 × 10^− 1^	†1.2 (0.1–13.3)	0.6 (0.1–2.3)
	NFE/ Tertiary	31 (8.2)	8.5 × 10^− 1^	†1.7 (0.7–3.9)	†1.0 (0.6–1.6)	178 (9.0)	9.5 × 10^− 1^	†1.8 (0.2–21.5)	0.9 (0.2–4.1)
5.	**# of children [2–4/ 1]**	17/ 362	1.3 × 10^− 1^	0.8 (0.4–1.4)	0.8 (0.6–1.1)	76/ 1,892	9.6 × 10^− 1^	0.05 (0.03–2.1)	0.05 (0.03–2.0)
6.	**Occupation**								
	Artisan/ Civil Servant	228/ 11	**1.0 × 10** ^**− 4**^	0.2 (0.08–0.4)	0.1 (0.07–0.2)	1,082/ 106	7.9 × 10^− 2^	0.4 (0.1–1.5)	0.5 (0.2–1.0)
	Casual labour/ Civil Servant	59 (15.6)	**1.0 × 10** ^**− 5**^	0.2 (0.07–0.3)	0.2 (0.1–0.3)	190 (9.6)	9.5 × 10^− 2^	0.3 (0.05–1.9)	0.4 (0.1–1.2)
	Unemployed/ Civil Servant	81 (21.4)	**1.0 × 10** ^**− 5**^	0.3 (0.1–0.7)	0.2 (0.1–0.4)	590 (29.9)	**2.2 × 10** ^**− 5**^	0.1 (0.04–0.4)	0.09 (0.04–0.2)
7.	**AMI (in $)**								
	101–200/ < 100	37/ 254	**1.0 × 10** ^**− 5**^	0.9 (0.6–1.5)	0.6 (0.4–0.7)	191/ 1,465	**2.0 × 10** ^**− 4**^	0.2 (0.04–0.9)	0.2 (0.06–0.4)
	201–300/ < 100	43 (11.4)	**1.0 × 10** ^**− 5**^	0.6 (0.3–1.0)	0.4 (0.3–0.5)	166 (8.4)	**3.0 × 10** ^**− 4**^	0.1 (0.01–0.9)	0.02 (0.01–0.1)
	> 400/ < 100	45 (11.9)	**3.0 × 10** ^**− 4**^	0.4 (0.3–0.7)	0.2 (0.1–0.3)	146 (7.2)	**2.0 × 10** ^**− 4**^	0.1 (0.01–1.1)	0.01 (0.001–0.1)
	**CONSTANT**		**1.0 × 10** ^**− 5**^				1.5 × 10^− 1^		

Legend. #; number, %; proportion of respondents, †; Most likelihood category, 95% C.I; 95% Confidence interval, AMI; Average Monthly Income, aOR; Adjusted odds ratio, Boldface numbers indicate significant *p*-values, *n*; frequency/count, NFE; No Formal Education, OR; Odds ratio, Reference Category; No

### Rate of RI during the COVID-19 pandemic

DPT/Penta3 (Diphtheria-Tetanus-Pertussis 3rd dose): The year 2019 and 2020–2020 (the same year) covered Diphtheria, Tetanus and Pertussis 3rd dose (Penta3) with an occupancy rate of 98 and 91, respectively. The decline in routine immunisation coverage in Rwanda was due to multiple factors, such as parental fear of infection at clinics, travel restrictions, and misinformation.

However, this study aimed at highlighting the impacts of COVID-19 on routine immunisation rather than the immunisation rates.

### Effect of misinformation on the continuity of routine immunisation

On misinformation about COVID-19 vaccines, 1,563 (76.4%, 95% C.I; 74.5–78.2) admitted to having received or heard negative information about the vaccines.

Logistic regression analysis revealed that except for age, all the demographic characteristics of respondents were significantly associated with, COVID-19 misinformation. Age, marital status, and monthly income were also found to be significantly associated with the trust of COVID-19 misinformation and the effect of COVID-19 on RI (Table 6).

**Table 6:** Misinformation, trust in misinformation and effect of the COVID-19 pandemic on RI

S/*N*	Characteristic	COVID-19 misinformation (*n* = 1,563)			Trust COVID-19 misinformation (*n* = 1,802)			Effect of COVID-19 on RI (*n* = 1,941)		
		*n* (%)	*p*-value	OR (95% C.I)	*n* (%)	*p*-value	OR (95% C.I)	*n* (%)	*p*-value	OR (95% C.I)
1.	**Age (in years)**									
	31–40/ ≤ 30	173/ 181	5.6 × 10^− 1^	†1.1 (1.0–1.3)	750/ 971	**1.0 × 10** ^**− 5**^	0.6 (0.5–0.7)	1,026/ 820	**3.3 × 10** ^**− 2**^	†1.3 (1.0–1.6)
	≥ 41/ ≤ 30	25 (6.6)	9.1 × 10^− 1^	†1.0 (0.6–1.7)	82 (4.6)	**1.9 × 10** ^**− 3**^	0.5 (0.4–0.8)	99 (4.8)	6.1 × 10^− 2^	0.5 (0.3–1.0)
2.	**Religion**									
	Protestant/Catholic	353/ 26	**5.0 × 10** ^**− 2**^	†1.8 (0.8–3.3)	1,727/ 76	**2.2 × 10** ^**− 2**^	†1.6 (1.1–2.4)	1,861/ 80	**< 0.001**	0.3 (0.2–0.5)
3.	**Marital status**									
	Single/Married	63/ 291	7.6 × 10^− 1^	0.9 (0.7–1.3)	324/ 1,408	6.9 × 10^− 2^	0.8 (0.6–1.0)	351/ 1,513	2.2 × 10^− 1^	†1.2 (0.8–1.6)
	Divorced/Married	18 (4.7)	**5.0 × 10** ^**− 4**^	†2.7 (1.5–4.7)	55 (3.1)	2.9 × 10^− 1^	0.7 (0.4–1.3)	58 (3.0)	**4.3 × 10** ^**− 2**^	0.4 (0.1–1.2)
	Widowed/Married	7 (1.8)	9.1 × 10^− 1^	†1.0 (0.4–2.6)	16 (0.9)	**1.0 × 10** ^**− 5**^	0.3 (0.1–0.4)	19 (1.0)	**1.0 × 10** ^**− 5**^	†5.9 (3.3–10.6)
4.	**Education**									
	Secondary/Tertiary	158/ 17	**5.0 × 10** ^**− 2**^	†1.9 (0.9–3.6)	762/ 83	9.4 × 10^− 1^	0.03 (0.01–0.1)	797/ 83	9.5 × 10^− 1^	†3.6 (2.6–4.5)
	Primary/Tertiary	173 (45.6)	1.6 × 10^− 1^	†1.6 (0.8–3.2)	817 (45.3)	9.3 × 10^− 1^	0.04 (0.02–0.2)	906 (46.7)	9.5 × 10^− 1^	†3.5 (3.1–5.3)
	NFE/ Tertiary	31 (8.2)	1.4 × 10^− 1^	†1.7 (0.8–3.7)	141 (7.8)	9.3 × 10^− 1^	0.03 (0.01–0.1)	155 (8.0)	9.4 × 10^− 1^	†1.2 (0.6–2.4)
5.	**# of children [2–4/ 1]**	17/ 362	8.9 × 10^− 1^	0.9 (0.5–1.6)	70/ 1,733	1.4 × 10^− 1^	†1.5 (0.9–2.7)	74/ 1,867	2.1 × 10^− 1^	0.6 (0.2–1.4)
6.	**Occupation**									
	Artisan/Civil Servant	228/ 11	9.5 × 10^− 2^	0.6 (0.4–1.1)	951/ 108	6.4 × 10^− 2^	0.4 (0.2–1.0)	1,047/ 110	9.4 × 10^− 1^	†4.3 (3.9–4.7)
	Casual labour/Civil Servant	59 (15.6)	**4.1 × 10** ^**− 2**^	0.5 (0.3–0.9)	187 (10.4)	1.6 × 10^− 1^	0.5 (0.2–1.3)	190 (1.8)	9.4 × 10^− 1^	†3.9 (3.4–4.3)
	Unemployed/Civil Servant	81 (21.4)	2.8 × 10^− 1^	0.7 (0.4–1.3)	557 (30.9)	3.6 × 10^− 1^	†1.5 (0.6–3.6)	594 (30.6)	9.5 × 10^− 1^	†2.3 (1.8–2.9)
7.	**AMI (in $)**									
	101–200/ < 100	37/ 254	1.4 × 10^− 1^	1.3 (0.9–1.9)	190/ 1,305	**1.0 × 10** ^**− 4**^	†9.9 (5.1–19.3)	192/ 1,436	**1.0 × 10** ^**− 5**^	0.1 (0.03–0.3)
	201–300/ < 100	43 (11.4)	**4.1 × 10** ^**− 2**^	†1.5 (1.0–2.3)	163 (9.0)	**1.0 × 10** ^**− 5**^	†9.3 (4.9–17.4)	166 (8.6)	**1.0 × 10** ^**− 5**^	0.1 (0.02–0.2)
	> 400/ < 100	45 (11.9)	**1.0 × 10** ^**− 5**^	†3.1 (2.0–4.8)	145 (8.0)	**1.0 × 10** ^**− 5**^	†29.5 (7.1–32.2)	147 (7.6)	9.2 × 10^− 1^	0.01 (0.00–0.1)
	CONSTANT		1.0 × 10^− 5^			9.6 × 10^− 1^			9.6 × 10^− 1^	

Legend. #; number, %; the proportion of respondents, †; Most likelihood category, 95% C.I; 95% Confidence interval, AMI; Average Monthly Income, Boldface numbers indicate significant *p*-values, *n*; frequency/count, NFE; No Formal Education, RI; Routine immunisation, OR; Odds ratio, Reference Category; Yes for COVID-19 misinformation/No for Trust COVID-19 misinformation/Yes for Effect of COVID-19 on RI

The factors affecting routine immunisation are presented in Table 7. Logistic regression analysis revealed that all the factors explored were significantly associated with RI. Culture, lack of funds hindering immunisation, trusting in misinformation, disagreeing on serious adverse effects of vaccines as well as disagreeing on serious adverse effects of COVID-19 vaccines were respectively; about seven times (6.8, 95% C.I:4.6–10.2), one and a half times (1.5, 95% C.I: 1.1–2.1), two and a half times (2.5, 95% C.I: 1.6–3.9), about four times (3.8, 9.5% C.I: 2.8–5.1) and about one and a third times (1.6, 95% C.I; 1.2–2.2) more likely to affect RI when compared with their various counterparts.

Of the 1,941 mothers who said the COVID-19 pandemic affected RI, 1,786 (92.0%), 1,778 (91.6%), 1,875 (96.6%), and 1,519 (78.3%) admitted that culture supports immunisation, the lack of funds to visit the health facilities hinders immunisation, trusted vaccine misinformation, and had concerns about the adverse effects of vaccines, respectively. Regarding the adverse effects of vaccines and COVID-19 vaccines, the mothers were about four times (OR; 3.8, 95% CI: 2.8–5.1), and one and a half times (OR; 1.6, 95% CI: 1.2–2.2) more likely to disagree that they are concerned with the serious adverse effects of vaccines in general and COVID-19 vaccines in particular when compared with having to agree on these adverse effects (Table 7).

**Table 7:** Factors affecting RI

S/*N*	Characteristic	Subclass	Effect of COVID-19 on RI (*n* = 1,941)			
			Yes (%)	No (%)	*p*-value	OR (95% C.I)
1.	Culture supports immunisation	Yes	87 (83.6)	1,786 (92.0)	**< 0.001**	†6.8 (4.6–10.2)
		No	17 (16.4)	155 (8.0)		
2.	Lack of funds hinders immunisation	Yes	69 (66.4)	1,778 (91.6)	**1.1 × 10** ^**− 2**^	†1.5 (1.1–2.1)
		No	35 (33.6)	163 (8.4)		
3.	Does vaccine misinformation affect your vaccine confidence? *	Yes	62 (62.0)	317 (16.6)	**< 0.001**	0.1 (0.1–0.2)
		No	38 (38.0)	1,597 (83.4)		
4.	Do you trust vaccine misinformation?	Yes	93 (89.4)	1,875 (96.6)	**< 0.001**	†2.5 (1.6–3.9)
		No	11 (10.6)	66 (3.4)		
5.	Concerned about adverse effects of vaccines	Disagree	43 (41.4)	422 (21,7)	**< 0.001**	†3.8 (2.8–5.1)
		Agree	61 (58.6)	1,519 (78.3)		
6.	Concerned about adverse effects of COVID-19 vaccines	Disagree	42 (40.4)	802 (41.3)	**1.2 × 10** ^**− 3**^	†1.6 (1.2–2.2)
		Agree	62 (59.6)	1,139 (58.7)		
7.	Concerned that COVID-19 vaccines might not be safe	Disagree	60 (57.7)	1,819 (93.7)	**< 0.001**	0.09 (0.07–0.1)
		Agree	44 (43.3)	122 (6.3)		
		Total	104	1,941		

Legend. %; proportion of respondents, †; Most likely category, 95% C.I; 95% Confidence interval, RI; Routine immunisation

## Discussion

Our study adds to the description of vaccine continuation and completion in Rwanda. This study revealed that; 94.1% of mothers obtained information about vaccines from health care workers, 5.5% had poor access to vaccines, 9.7% complained of lack of transport fare to the vaccination centre, 62.4% had a good perception of vaccine risks, 76.4% had been misinformed on COVID-19 vaccines, 96.2% trust vaccine misinformation, 88.1% trust COVID-19 vaccine misinformation, and 94.9% admitted that RI of their children was affected by the COVID-19 pandemic.

### Sources of information

We observed that the major source of information on vaccines and vaccination was provided by healthcare providers (94.1%), followed by the mass media and social media (28.8%) and relationships (7.1%). This was in line with previous studies in Nigeria, Cyprus and Switzerland, where parents rely on paediatricians and nurses for information concerning childhood vaccination [50–52], but differed from studies in the Netherlands, Philippines, and Guinea where parents mostly explored the internet, as well as relied on traditional authorities for information about childhood vaccinations [53–55]. In other studies, parents considered factors like access to information, interpersonal communication, misinformation, and community norms for childhood vaccination [56–59]. The differences in the rates and variety of sources of vaccine information could be explained by the fact that the present study was a nationwide-based study in which many sources of information on vaccines were explored amongst mothers.

### Reasons for vaccination/immunisation discontinuation

The 5.5% proportion of mothers who complained of inaccessibility to healthcare facilities in this study was low compared with a study on the concerns about COVID-19 accessibility, affordability, and acceptability [60]. Our results found inaccessibility to healthcare facilities as the barrier to immunization uptake, though this is not the case in focus group studies in Kenya and the Netherlands, where the significant factors were perceived risk of disease, perceived side effects of vaccines, negative past experiences of vaccinations, and social environmental influence [61, 62].

### Religious and traditional tendencies towards the immunisation of children

In our study, 92.2% and 91.6% of the mothers indicated that their religion and culture were in support of childhood vaccination and immunisation. In our study, religion was associated with age, marital status, education and average monthly income; which was synonymous with maternal education being associated with vaccination coverage in 127 countries worldwide [63]. The support of childhood immunisation by culture is in line with ethnic backgrounds as reported amongst Black and Asian minority ethnic groups in the United Kingdom [64].

### Mothers’ perception of risks

In our study, 77.3%, 58.7%, and 8.1% of the mothers respectively agreed to the fact that; there were serious AEs of vaccines, serious AEs of COVID-19 vaccines, and that the COVID-19 vaccines may not be safe. This was similar to the fear of vaccines, expressed by mothers in a study reported in Australia [65], the fear of COVID-19 vaccines due to history of COVID-19 infection in Nepal [66], and contrary to the perception of 91.7% of Greek mothers who believed that COVID-19 vaccines would protect their children [67]. The 91.9% rate of respondents in our study who agree that COVID-19 vaccines may be safe was less than the 45.3% reported among parents in Egypt [68]. The differences in the perception of either vaccine or COVID-19 vaccine risks may be due to the differences in the study populations as well as study designs.

### Vaccine misinformation

In this study, 18.5% of the mothers had been misinformed about vaccines in general, and 76.4% had been misinformed about COVID-19 vaccines. The 76.4% misinformation on COVID-19 vaccine in this study is more than the 57.6% in the USA [69]. These results suggest that people’s background and social conditions shape how they receive and believe vaccine information. Older or less educated mothers, those from certain religious groups, or those with lower income or informal jobs may have less access to accurate information and be more exposed to rumors or false claims about vaccines. Meanwhile, mothers with more education, higher income, or better access to health services tend to be better informed and less affected by misinformation.

### Factors affecting routine immunisation

The 94.9% who admitted that RI of their children worldwide had been disrupted during the COVID-19 pandemic reported that movement restrictions, fear of COVID-19 transmission, and misinformation contributed to missed or delayed vaccinations [70]. as well as the 96.3% of measles immunisation post-coverage reported in Nigeria [71]. This was however higher when compared with the 72% rate for DPT3 reported in Laos [72]; the 71–72% Measles-Rubella and yellow fever virus, 71% OPV3, 82.5% Penta 3, and 83.5% BCG reported in the rural settings of the Gambia and India [73, 74], as well as the 18.8–69.1% reported among Pakistani children [75]. The differences may be due to differences in study designs.

In our study, the following factors affected RI the lack of funds, COVID-19 vaccine misinformation, concern about the adverse effects of vaccines and COVID-19 vaccines, as well as the safety of COVID-19 vaccines. Our findings were different from; the willingness of mothers to pay for immunisation, distance to the immunisation, illiteracy, occupation, family size, home delivery and health education, as reported in Laos as well as the Gambia [72, 73]. Our findings were however similar to the educational level of parents, occupation and source of healthcare information among Pakistani parents [75].

### Global impacts of COVID-19 on routine immunisation

COVID-19 has caused substantial trouble to normal immunisation programs across the globe, including in Rwanda. Movement restrictions, fear of infection, and redistribution of healthcare resources to deal with the pandemic caused changes in previously planned children’s vaccinations or their delay. The disruptions created a reduction in the coverage of the major vaccines, and this risked the renewal of outbreaks of vaccine-preventable illnesses. In a number of studies, attendance in health facilities to be immunized has been reported to be on the decrease during high seasons of the pandemic. The knowledge of these effects highlights the need to have resilient healthcare policies and adaptative strategies that ensure the delivery of the necessary services in times of crisis.

### Practical significance of healthcare policy in child immunisation

The practical importance of healthcare policy in regard to child immunisation is that the policy has been found to guarantee equitable access, sustained coverage, and efficient delivery of the vaccination services. Effective policies can be used to direct the distribution of resources, strengthen supply chains and make immunisation a part of primary healthcare systems. These policies in Rwanda have facilitated a nationwide immunisation campaign, field work by health workers to communities, and early mortality surveillance, resulting in a high rate of immunisation and a decrease in child mortality. Healthcare policies give priority to equity, health education and sustainable funding, thus preventing not only the children against vaccine-preventable diseases but ensuring a better and stronger health system in general and long-term resiliency of the population in terms of health.

### Strengths and limitations

#### Strengths

The data collection was carried out by field staff who were familia with the local terrain, ensuring a thorough understanding of the study areas. The quality of the data collected was assured through pretesting of questionnaires in a pilot study. The pilot study aimed to minimise bias and errors, improving the accuracy and reliability of the data. The minimisation of bias was done by randomisation in the selection of Provinces, Districts, Sectors, Cells, and Villages, screening and excluding other caregivers who were not mothers, as well as sorting out and eliminating outliers. The study had significant strength in the large sample size and the use of the face-to-face method of administering questionnaires, which enhanced the response rate.

#### Limitations

This was a cross-sectional study, representing the snapshot of the population within the study period. Thus, we cannot infer causal relationships between mothers’ religion/culture, perception of risks, and continuity of childhood vaccination with their demographic characteristics. Data were collected through anonymous self-reporting via door-to-door, and thus there is a possibility of response bias and recall bias. Such biases can also affect some of the responses as well as the results of the study. Another significant limitation was representativeness as a higher proportion of the sampled respondents were undereducated, having acquired less than the secondary level of education.

## Conclusion

The COVID-19 pandemic negatively affected routine child immunisation globally and in Rwanda, causing temporary declines in vaccination coverage due to service disruptions, movement restrictions, and parental concerns. These findings emphasise the need for robust contingency plans, community education, and resilient healthcare resources to ensure that essential child immunisation programmes remain unaffected during future health emergencies.

## Supplementary Information

Below is the link to the electronic supplementary material.

Supplementary Material 1: Supplementary file 1. Survey Questionnaire

Supplementary Material 2: Supplementary file 2. Supplementary Table

## Data Availability

The data supporting this study’s findings are available from the corresponding author upon reasonable request.

## Abbreviations

$$\bar{\mathrm{x}}$$: Mean
95% C.I: 95% Confidence Interval
AEs: Adverse effects
aOR: Adjusted Odds Ratio
BCG: Bacille Calmette-Guérin
COVID-19: Coronavirus Disease 2019
DTP3: 3rd dose Diphtheria, Tetanus Toxoid and Pertussis
MCV1: 1st dose of Measles Containing Vaccine
OPV3: 3rd dose of Oral Poliovirus Vaccine
OR: Odds Ratio
RI: Routine Immunisation
VH: Vaccine hesitancy
χ^2^: Pearson Chi square
