# Supplementary material for: The impacts of COVID-19 on routine immunization for children in Rwanda
Source: BMC Infect Dis. 2026 Jan 24;26:275. doi: 10.1186/s12879-026-12623-0 (PMC12879442; doi:10.1186/s12879-026-12623-0)
Supplement: Supplementary file 2 — Supplementary Material 2: Supplementary file 2. Supplementary Table [file 12879_2026_12623_MOESM2_ESM.docx]

**Supplementary Data**

Supplementary Table 1: Sources of child vaccination information

| Source | Frequency (%) | 95% C.I |
| --- | --- | --- |
| Friends, co-workers, or neighbours | 145 (7.1) | 6.1 - 8.3 |
| Traditional Leaders | 167 (8.2) | 7.1 - 9.4 |
| Religious Leaders | 212 (10.4) | 9.1 – 11.8 |
| Social media (Facebook, Twitter, WhatsApp) | 251 (12.3) | 10.9 – 13.8 |
| Mass Media (Radio, Television) | 589 (28.8) | 26.9 - 30.8 |
| Health workers (Nurses/ Doctors) | 1,925 (94.1) | 93.0 - 95.1 |

Supplementary Table 2: Reasons for not taking children for vaccinations

| Most important reason for not taking child to vaccinate | Frequency (%) | 95% C.I |
| --- | --- | --- |
| Inaccessible Health Centre | 122 (5.5) | 4.6 – 6.6 |
| I don’t belief the vaccines are good for my child(ren) | 44 (2.0) | 1.5 – 2.7 |
| My child was not born in the hospital | 48 (2.4) | 1.8 – 3.1 |
| Other reasons | 1,844 (90.2) | 88.8 – 91.4 |
| *Total* | 2,045 |  |
| Other Reasons |  |  |
| I don’t have a problem | 1,781 (96.6) | 95.6 – 97.3 |
| Ignorance | 30 (1.6) | 1.1 – 2.3 |
| My child is fully immunised | 21 (1.1) | 0.7 – 1.7 |
| Poor communication | 12 (0.6) | 0.4 – 1.1 |
| *Total* | 1,844 |  |

| Does lack of funds hinder child immunisation? | Frequency (%) | 95% C.I |
| --- | --- | --- |
| Yes | 198 (9.7) | 8.5 – 11.0 |
| No | 1,847 (90.3) | 89.0 – 91.5 |
| *Total* | 2,045 |  |

Supplementary Table 3: Association of lack of funding to health care facility with occupation and monthly income

| Variable | Subclass | No | Yes (%) | Total (%) | χ^2^ | *p* - value |
| --- | --- | --- | --- | --- | --- | --- |
| Occupation | Artisan | 995 | 148 (74.75) | 1,143 (55.89) | 35.864 | **< 0.001** |
|  | Casual labour | 175 | 17 (8.59) | 192 (9.39) |  |  |
|  | Civil Servant | 108 | 2 (1.01) | 110 (5.38) |  |  |
|  | Unemployed | 569 | 31 (15.66) | 600 (29.34) |  |  |
| AMI (in $) | < 100 | 1,353 | 185 (93.43) | 1,538 (75.21) | 45.261 | **< 0.001** |
|  | 101 – 200 | 180 | 13 (6.57) | 193 (9.44) |  |  |
|  | 201 – 300 | 167 | 0 (0.0) | 167 (8.17) |  |  |
|  | > 400 | 147 | 0 (0.0) | 147 (7.19) |  |  |
| Total |  | 1,847 | 198 | 2,045 |  |  |

Supplementary Table 4: Reasons for vaccine dose incompletion

| Most important reason for vaccine dose incompletion | Frequency | 95% C.I |
| --- | --- | --- |
| One neighbour's child got some health complications attributed to the vaccine shots. | 4 (0.2) | 0.08 - 0.50 |
| I don't think these vaccines are good for my child | 99 (4.8) | 4.0 - 5.9 |
| The child is healthy and I feel there is no need to continue the doses. | 99 (4.8) | 4.0 - 5.9 |
| Others | 1,843 (90.1) | 88.7 - 91.3 |
| *Total* | **2,045** |  |
| Other Reasons | **Frequency** | **95% C.I** |
| No funds to reach health centre | 1 (0.1) | 0.01 - 0.3 |
| Everyone should vaccinate the child | 3 (0.2) | 0.06 - 0.5 |
| Laziness | 5 (0.3) | 0.12 - 0.6 |
| Continue other dose | 10 (0.5) | 0.3 - 1.0 |
| I missed the date | 14 (0.8) | 0.4 - 1.3 |
| My child completed the immunisation doses | 20 (1.1) | 0.7 - 1.7 |
| There's no reason for not vaccinating my child completely | 1,790 (97.1) | 96.3 - 97.8 |
| *Total* | **1,843** |  |
